# Trifostigmanoside I, an Active Compound from Sweet Potato, Restores the Activity of MUC2 and Protects the Tight Junctions through PKCα/β to Maintain Intestinal Barrier Function

**DOI:** 10.3390/ijms22010291

**Published:** 2020-12-30

**Authors:** Amna Parveen, Seungho Choi, Ju-Hee Kang, Seung Hyun Oh, Sun Yeou Kim

**Affiliations:** College of Pharmacy, Gachon University, 191 Hambakmoero, Yeonsu-gu, Incheon 21936, Korea; amnaparvin@gmail.com (A.P.); schoi790227@gmail.com (S.C.); applekjh0503@hanmail.net (J.-H.K.)

**Keywords:** tight junctions, sweet potato extract, protein kinase C, mucin

## Abstract

Sweet potato (*Ipomoea batata*) is considered a superfood among vegetables and has been consumed for centuries. Traditionally, sweet potato is used to treat several illnesses, including diarrhea and stomach disorders. This study aimed to explore the protective effect of sweet potato on intestinal barrier function, and to identify the active compounds of sweet potato and their underlying mechanism of action. To this purpose, bioactivity-guided isolation, Western blotting, and immunostaining assays were applied. Interestingly, our bioactivity-guided approach enabled the first isolation and identification of trifostigmanoside I (TS I) from sweet potato. TS I induced mucin production and promoted the phosphorylation of PKCα/β in LS174T human colon cancer cells. In addition, it protected the function of tight junctions in the Caco-2 cell line. These findings suggest that TS I rescued the impaired abilities of MUC2, and protected the tight junctions through PKCα/β, to maintain intestinal barrier function.

## 1. Introduction

Disturbances of gastrointestinal functions, such as constipation, diarrhea, obesity, ulcerative colitis (UC), irritable bowel syndrome (IBS), and inflammatory bowel diseases (IBD), affect millions of people worldwide, thereby inducing a decrease in their quality of life as well as a considerable financial burden [1]. The enormous surface area of the gastrointestinal tract not only efficiently absorbs electrolytes, water, and nutrients from food, but it also provides a consistent barrier against pathogens and harmful compounds from the external environment. This intestinal barrier is mainly composed of the mucosal layer, epithelial layer, and lamina propria [2]. The interplay between these layers in terms of regulation of structural components and molecular interactions contributes in maintaining the intestinal barrier function [3]. In particular, specialized epithelial cells secrete mucin (MUC2), a polypeptide that forms a hydrated gel to cover the mucosal surfaces. Mucins prevent large particles from directly contacting the epithelial cell layer [4]. Animal studies revealed that MUC2 deficiency leads to gastrointestinal tract inflammation, gross bleeding, severe growth retardation, and increased permeability. In addition, tight junctions (TJs), complex protein systems composed of the transmembrane proteins claudins and occludins, interact with zonula occludens (ZO) proteins that regulate the paracellular permeability of the intestinal epithelial barrier. Therefore, intracellular TJs and intestinal mucus are prominent elements that work together in maintaining the intestinal barrier function, and thus are putative therapeutic targets for gastrointestinal disorders. However, the complex mechanism and crosstalk between disease-promoting factors pose significant limitations to therapy for gastrointestinal diseases. Therefore, the scientific community is trying to identify alternative therapies to mitigate the various etiologic factors of intestinal disorders through the modulation of different signaling pathways. Interestingly, complementary approaches based on traditional medicine have attracted the attention of researchers for the recovery of patients with intestinal disorders owing to the presence of various phytochemicals.

Sweet potato (*Ipomoea batata*) is considered a superfood among vegetables and has been consumed for centuries. This starchy, large, and tuberous root vegetable, belonging to the family Convolvulaceae, has been very useful to mankind. For instance, many species of this genus are routinely used for religious rituals, as well as for ornamental and medicinal purposes. Moreover, sweet potato is considered a healthy food and has recently attracted great attention from consumers, food industries, and the scientific community, owing to the presence of several active secondary metabolites, including nutraceutical components, carotenoids, anthocyanins, flavonoids, and coumarins. Traditionally, sweet potato is consumed to treat several diseases, including diarrhea and stomach disorders. However, the role of this vegetable in the treatment of gastrointestinal disorders has not yet been explored by scientific investigation [5]. Therefore, this study was specifically designed to scientifically demonstrate the effectiveness of the traditional use of sweet potato in controlling gastrointestinal disorders. In particular, we examined the efficacy of sweet potato extracts (SPEs) in improving intestinal barrier function, and we identified potential bioactive components and the signaling pathways that they modulate.

## 2. Results and Discussion

### 2.1. Isolation, Characterization, and Standardization of Compound A

**Figure 1:**
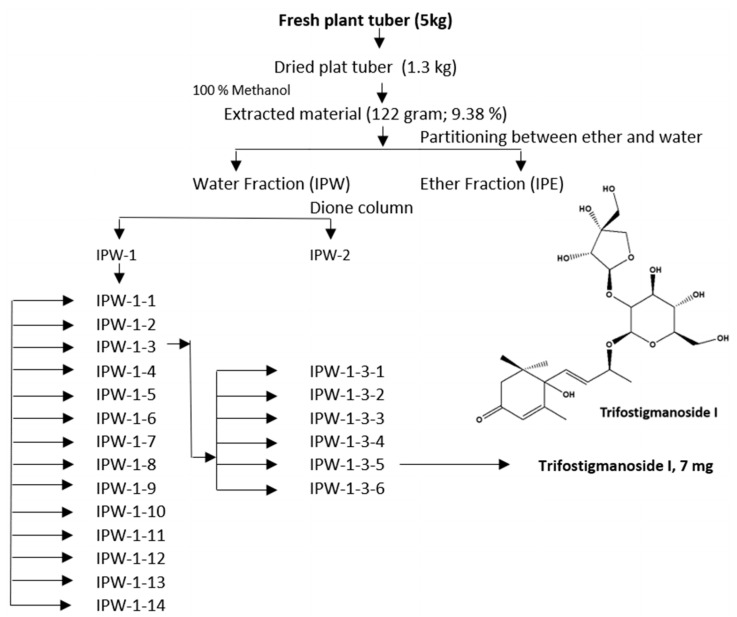
Isolation scheme of trifostigmanoside I from sweet potato extract.

**Figure 2:**
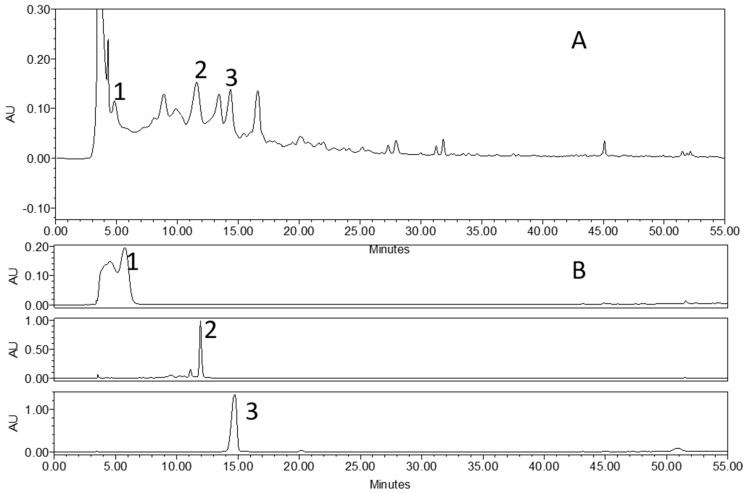
HPLC chromatograms of sweet potato extract and individual compounds: (**A**) Chromatogram of sweet potato extract; (**B**) chromatogram of standard compounds: (1) scopolin, (2) trifostigmanoside I, and (3) scopoletin.

Bioactivity-guided isolation using consecutive chromatography on a Sephadex LH-20 column resulted in the isolation of an active compound, as shown in Figure 1. After isolation, the compound was recrystallized for purity. Compound A was identified through ^1^H NMR, ^13^C NMR, and mass spectroscopy and found to possess the molecular formula C_24_H_38_O_12_. HR-FAB-MS (positive) *m*/*z*: 541.2261 [M+Na]+ C12H38NaO12, 387 [M-H-Api]+,225 [M-H-Api-glc]+. ^1^H NMR (600 MHz, CD3OD): δ: 5.87 (1H, d, J = 15.6, H-7), 5.86 (1H, d, J = 15.6, H-8), 5.85 (1H, s, H-4), 5.37 (1H, d, J = 1.4, H-1″), 4.44 (1H, m, H-9), 4.04 (1H, d, J = 7.7, H-1′), 2.51 (1H, d, J = 16.5, H-2a), 2.16 (1H, d, J = 16.5, H-2b), 1.92 (3H, s, H-13), 1.29 (3H, d, J = 6.4, H-10), 1.04, (3H, s, H-12), 1.03 (3H, s, H-11). ^13^ CNMR (600MHz, CD3OD): 41.02 (C-1), 50.7 (C-2), 199.7 (C-3), 125.7 (C-4), 165.8 (C-4), 77.7 (C-5), 130.31 (C-6), 133.3 (C-7), 133.3 (C-8), 75.4 (C-9), 19.70 (C-10), 22.02 (C-11), 23.32 (C-12), 18.13 (C-13), 101.3 (C-1′), 77.2 (C-2′), 75.4 (C-3′), 64.7 (C-4′), 73.4 (C-5′), 61.3 (C-6′), 109.3 (C-1″), 76.5 (C-2″), 79.2 (C-3″), 59.6 (C-4″), 70.2 (C-5″). Therefore, compound A was identified as trifostigmanoside I [6]. The purity of trifostigmanoside I, as investigated using HPLC, was found to be 98%. HPLC-UV analysis at 254 nm revealed the major constituents of SPEOW, and the observed peaks were assigned to (1) scopolin, (2) scopoletin, and (3) trifostigmanoside I. Their retention times in the chromatogram were compared with those of standard compounds, as illustrated in Figure 2. As a result, the contents of scopolin, trifostigmanoside I, and scopoletin were found to be 5.84, 5.48, and 5.27 µg, respectively, per 100 mg of powdered *I. batata*.

### 2.2. Trifostigmanoside I Promotes Mucin Expression

**Figure 3:**
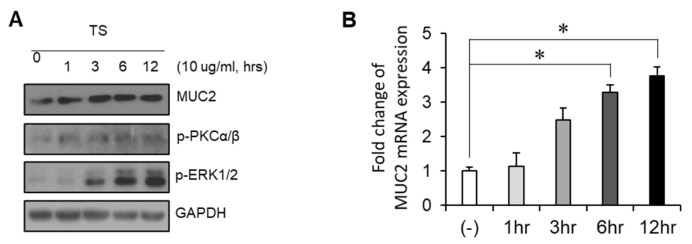
Identification of the signaling pathway involved in trifostigmanoside I-induced MUC2 expression. (**A**) LS174T cells were treated with trifostigmanoside I at different time points, and the expressions of MUC2, p-PKCα/β, p-ERK1/2, and GAPDH were assessed by Western blotting. (**B**) Semi-quantitative real-time PCR to assess the expression of MUC2 in LS174T cells after treatment with trifostigmanoside I at different time points. Data are presented as mean ± SEM of triplicate assays. All Western blot results were quantified by using Image J. * *p* < 0.05. TS, trifostigmanoside I.

**Figure 4:**
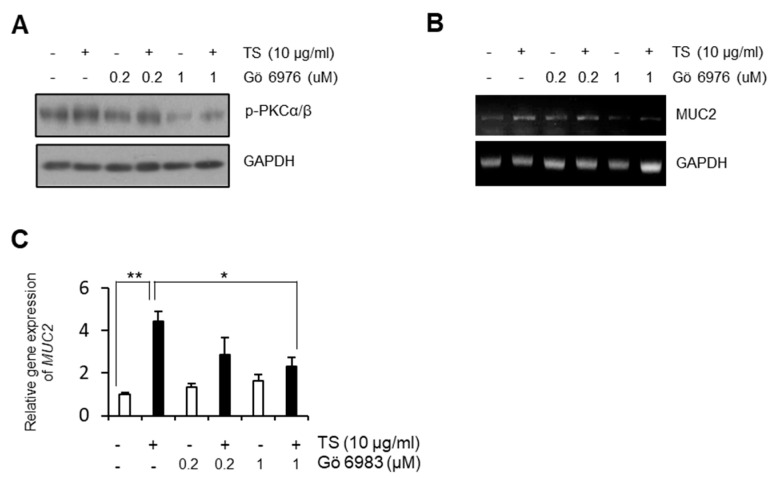
Effect of PKC inhibitors on the impact of trifostigmanoside I on LS174T cells. The PKC inhibitor suppressed TS-dependent MUC2 expression. (**A**) Expression of p-PKCα/β and GAPDH in LS174T cells after trifostigmanoside I treatment with or without Gö6976. (**B**) mRNA expression of MUC2 in LS174T cells after trifostigmanoside I treatment with or without Gö6976. (**C**) Semi-quantitative real-time PCR to assess the effect of pan-PKC inhibitor, Gö6983, on MUC2 expression. Data are presented as mean ± SEM of triplicate assays. All Western blot results were quantified by using Image J. * *p* < 0.05, ** *p* < 0.01. TS, trifostigmanoside I.

In order to investigate the effect of trifostigmanoside I on MUC2 expression, LS174T cells were treated with trifostigmanoside I, and Western blotting and semi-quantitative real-time PCR were performed. As shown in Figure 3, trifostigmanoside I treatment significantly promoted the expression of MUC2 in LS174T cells in a time-dependent manner, at both transcriptional and protein levels. In previous reports, MUC2 expression was found to be induced via activation of PKCα/β and its downstream molecule, ERK1/2 [7,8]. Therefore, we examined changes in the activation of PKCα/β-ERK1/2 signaling using Western blotting. Notably, treatment with trifostigmanoside I markedly increased the phosphorylation of PKCα/β and ERK1/2 as well as the expression of MUC2 (Figure 3, Supplementary Figure S1). To investigate whether increased MUC2 expression was due to activation of PKCα/β-ERK1/2, the most potent PKC inhibitor targeting PKCα/β, GÖ6976, was used for further experiments. Consistent with the previous experiment, treatment of LS174T cells with trifostigmanoside I promoted PKCα/β phosphorylation (Figure 4A) and MUC2 expression (Figure 4B). On the other hand, combined treatment with trifostigmanoside I and GÖ6976 reduced the phosphorylation of PKCα/β (Figure 4A, Supplementary Figure S2) and prevented MUC2 expression (Figure 4B). We assessed the effect of another PKC inhibitor, Gö 6983, which is a pan-PKC inhibitor, on the expression of MUC2 as cells were treated with TS. As expected, Gö 6983 also inhibited mucin expression (Figure 4C). These results indicate that trifostigmanoside I promoted the expression of MUC2 by activating PKCα/β-ERK1/2 signaling.

### 2.3. Sweet Potato Extract Does not Affect Lipopolysaccharide-Induced Inflammatory Response

**Figure 5:**
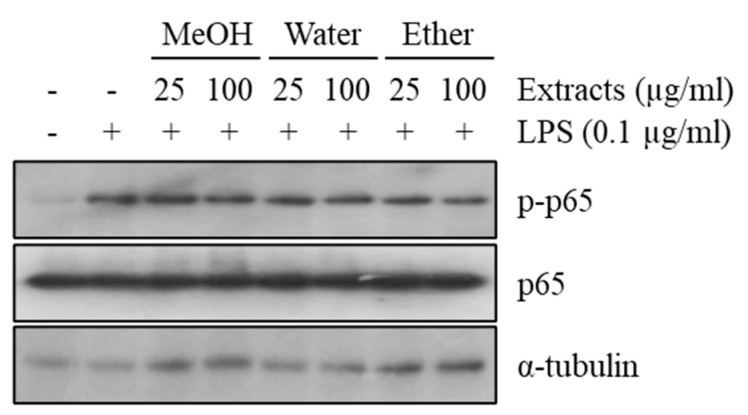
Effect of sweet potato extracts on p65 phosphorylation in the Raw264.7 mouse macrophage cell line. Raw264.7 cells were pre-treated with sweet potato MeOH, water, or ether extract and then treated with LPS. Phosphorylation of p65 was assessed by Western blotting. All Western blot results were quantified by using Image J.

Since IBD is known as a chronic inflammatory disease [9], the anti-inflammatory effect of various SPEs was examined. Raw264.7 cells, a murine macrophage cell line, were pre-treated with the extracts at the indicated concentrations, and further treated with lipopolysaccharide (LPS). As shown in Figure 5, LPS induced the phosphorylation of p65. However, pre-treatment with water extract, which includes TS, did not prevent the induction of p65 phosphorylation (Figure 5, Supplementary Figure S3). In addition, MeOH and ether extracts did not block P65 activation, indicating that SPEs may not have anti-inflammatory properties (Figure 5).

### 2.4. Effect of Sweet Potato Extracts on the Function of Tight Junctions in Caco-2 Cells

**Figure 6:**
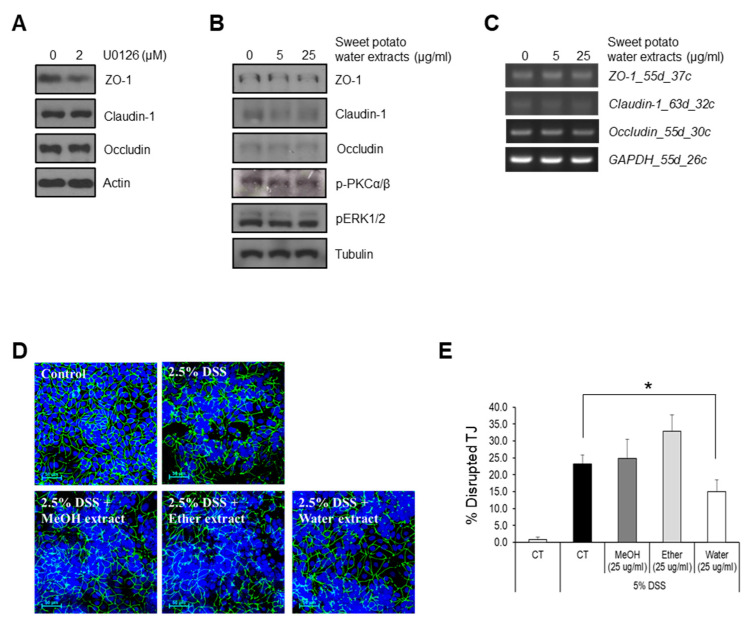
Effect of sweet potato extracts on tight junctions in Caco-2 cells. The effect of sweet potato water extract on tight junctions was assessed. (**A**) Caco-2 cells were treated with ERK inhibitor, U0126, and the expression of tight junction-related proteins was assessed by Western blotting. Caco-2 cells were treated with sweet potato water extract with the indicated concentrations, and the expression of tight junction-related proteins was assessed by Western blotting (**B**) and RT-PCR (**C**). (**D**) Caco-2 cells were treated with 2.5% DSS +/− MeOH, ether, or water extracts. Immunofluorescence assay for ZO-1 was performed to assess the orientation of tight junctions. Scale bar = 50 µm. (**E**) Quantification of immunofluorescence assay. Percent disrupted tight junctions was calculated by dividing the number of ZO-1-disrupted (wrinkled or discontinued) cells by the total number of cells in high-power fields. Data are presented as mean ± SEM of three independent experiments. All Western blot results were quantified by using Image J. * *p* < 0.05.

To determine whether SPEs exhibit a protective effect against TJs disruption by dextran sulfate sodium (DSS), the intestinal epithelial cell line Caco-2 was used. It has been reported that the orientation and expression of TJs proteins are regulated by the MAPK/ERK signaling pathway [10]. Our results show that inhibition of ERK activity by U0126 decreased the expression of ZO-1, a major component of TJs (Figure 6A, Supplementary Figure S4A). Since TS contributed to activation of ERK signaling, as shown in Figure 3A, we examined if water SPE could induce the expression of TJ proteins. Unexpectedly, there was no effect on promotion of the expression of TJ proteins (Figure 6B,C, Supplementary Figure S4B). However, we found that water SPE protected intestinal epithelial cells from DSS damage (Figure 6D,E). Caco-2 cells were polarized for 2 weeks, treated with the extracts at indicated doses for 24 h, and then further treated with 2.5% DSS for 48 h. Cells were then subjected to an immunofluorescence assay for the detection of ZO-1, a protein localized on TJs. Normal Caco-2 cells exhibited a smooth TJ network without cuts or wrinkles (Figure 6D,E). On the other hand, DSS-treated Caco-2 cells displayed TJs with impaired orientation, leading to a discontinuous or wrinkled TJs network (Figure 6D,E). Pre-treatment with MeOH or ether extracts did not protect TJs from DSS-mediated damage. On the other hand, pre-treatment with water SPE at a high dose partially rescued TJs damage (Figure 6D,E). These results indicate that water SPE played a protective role against TJ damage. However, since water SPE did not affect the expression levels of TJ proteins, there could be another pathway that protects TJs other than the ERK signaling pathway.

## 3. Materials and Methods

### 3.1. Preparation of I. Batata Extract

Sweet potatoes were purchased from an open market (Incheon, Korea). Tubers (5 kg) were cut, dried under shade, and powdered. Next, sweet potato extract (SPE) was extracted with 100% methanol for 72 h. SPE was then filtered, concentrated under vacuum, and dried to powder using a freeze dryer. The dry extract yield was 9.38%. The extract was stored at −80 °C until further analysis.

### 3.2. Bioactivity-Guided Isolation of Active Compounds from I. Batata Extract

The dry extract (120 g) was partitioned between ether (11 g) and water (100 g) solutions. A bioactivity-guided approach led to the selection of the water extract for further processing. This was loaded onto a Dione column to separate the non-sugar fraction (IPW-1) from the sugar fraction (IPW-2). Next, a Sephadex LH-20 open column was used to isolate active compounds from IPW-1 within a gradient mobile phase (10–100%) of methanol, and 14 fractions were obtained. Repeated column chromatography and bioactivity assays led to the isolation and purification of compound A. Detailed isolation schemes are shown in Figure 1. Compound A was identified using ^1^H nuclear magnetic resonance (NMR), ^13^C NMR, and mass spectroscopy.

### 3.3. Standardization of I. Batata Extract

High-performance liquid chromatography (HPLC) was performed using a Waters system (Waters Corp., Milford, MA, USA). For quantitative analysis, a sample was prepared by collecting 500 mg of total extract and removing sugar with a Dione column; the sugar-depleted extract, named sweet potato extract with sugar (SPEOW), was then dried (12 mg), dissolved in 1 mL of methanol, and filtered through a membrane filter (pore size, 0.22 µm) before HPLC analysis. A 20 µL sample was injected into a C18 Phenomax column (250 × 4.6 mm; particle size, 5 µm). The column was maintained at room temperature. The gradient mobile phase consisted of 30% ammonium acetate (50 mM, pH 4.5) for the first 2 min and 30–80% methanol until 40 min. For standardization, sugar was removed from SPE using a Dione column. The remaining extract was dried at a concentration of 10 mg/mL. Scopolin, compound A, and scopoletin were quantified using integration of peak areas measured at 254 nm from four different concentrations.

### 3.4. Cell Culture and In Vitro Experiments

All cell lines were incubated at 37 °C in a humidified chamber with 5% CO_2_. The human intestinal epithelial cell lines LS174T and Caco-2 were purchased from the American Type Culture Collection (ATCC, Rockville, MD, USA). To investigate the effect of SPE on mucin expression, the LS174T cell line was used. LS174T cells were cultured in RPMI-1640 (WelGene, Daegu, Korea) supplemented with 10% fetal bovine serum (FBS, WelGene, Daegu, Korea) and streptomycin (100 µg/mL)/penicillin (100 units/mL). Cells (5 × 10^5^ cells/well) were seeded on six-well plates and incubated for 24 h. First, to investigate the effect of SPE on mucin expression, cells were treated with PW9-3 (10 μg/mL) and incubated for 1, 3, 6, and 12 h. In a second assay, LS174T cells were treated with PW9-3 (10 μg/mL) and GÖ6976 or GÖ6983 (Calbiochem-Novabiochem, Alexandria, New South Wales, Australia) was treated twice every 12 h, for a total incubation of 24 h since the first treatment. Total protein and RNA were isolated and subjected to Western blot and semi-quantitative real-time PCR, respectively.

To examine the effect of SPE on TJs, the Caco-2 cell line was used. Caco-2 cells were seeded on coverslips inserted in the wells of a 12-well plate. Cells were incubated until they reached 100% confluence, and further incubated for 2 more weeks to induce polarization. Cells were then pre-treated with MeOH, ether, or water SPEs (25 or 50 μg/mL) for 24 h. Next, the medium was changed to a fresh medium containing the same extracts together with 2.5% dextran sulfate sodium (DSS) to induce damage to the TJs. Cells were incubated for 48 h and then subjected to immunofluorescence assay.

### 3.5. Western Blotting

In order to isolate total proteins, cells were lysed in RIPA lysis buffer, which consisted of 20 mM Tris-HCl (pH 7.6), 100 mM NaCl, 1 mM EDTA, 1% NP-40, 0.5% sodium deoxycholate, 1% Triton X-100, 1 mM sodium fluoride, 1 mM sodium vanadate, and protease inhibitor cocktail (Roche Diagnostics, Mannheim, Germany). Protein concentration was measured using a bicinchoninic acid protein assay kit (Pierce, IL, USA). Prior to Western blotting, protein samples were denatured by boiling for 5 min at 95 °C in a sample buffer containing 2% sodium dodecyl sulfate (SDS), 60 mM Tris (pH 6.8), 25% glycerol, 5% 2-mercaptoethanol, and 0.1% bromophenol blue. SDS-polyacrylamide gel electrophoresis (PAGE) was performed using 20 μL of each protein sample. The proteins were transferred to polyvinylidene fluoride membranes (PALL Life Science, New York, NY, USA), which were then blocked with TBS supplemented with 0.1% Tween-20 and 5% skim milk. Next, the membranes were incubated with primary antibodies overnight at 4 °C and then with secondary antibodies for 1 h at room temperature. Antibody-conjugated proteins were detected using an Absignal kit (Abclone, Seoul, Korea) and an enhanced chemiluminescence (ECL) substrate. All Western blot experiments were independently performed three times, and results were quantified using Image J.

### 3.6. Reverse Transcription- and Semi-Quantitative Real-Time PCR

RNA was isolated from cells using TRIzol reagent (Invitrogen, Carlsbad, CA, USA) as described in the manufacturer’s protocol. Two micrograms of RNA was used to synthesize cDNA using a PrimeScript RT reagent Kit (Takara, Shiga, Japan), following the manufacturer’s protocol. Reverse-transcription PCR (RT-PCR) and semi-quantitative real-time PCR were performed using the primers listed in Table 1. Agarose gel electrophoresis was performed to detect the RT-PCR products. To quantify gene expression levels, semi-quantitative real-time PCR was performed. Relative gene expression analysis was conducted using the method described in a previous report [11]. Each sample was analyzed in triplicate.

### 3.7. Immunofluorescence Assay

To observe TJs in Caco-2 cells, ZO-1 expression was investigated using an immunofluorescence assay. Cells were first fixed in a MeOH:acetone mixture (1:1) at −20 °C for 20 min, and subsequently blocked with Antibody Diluent Reagent (Invitrogen) at room temperature for 30 min. Cells were then incubated with a rabbit anti-ZO-1 antibody (Invitrogen; 1:1000) at 4 °C overnight. Cells were washed with PBS-T (supplemented with 0.1% Tween-20) three times and incubated with an anti-rabbit IgG-FITC antibody (Santa Cruz Biotechnology, Dallas, TX, USA; 1:500) at room temperature for 2 h. Next, cells were washed with PBS-T three times and mounted on coverslips with a DAPI mounting medium (Vector Laboratories Inc., Burlingame, CA, USA). Finally, stained cells were observed using a confocal microscope (Nikon Instruments Inc., New York, NY, USA).

## 4. Discussion

It is well acknowledged that disturbed gastrointestinal functions pose a considerable burden on health care worldwide, leading to a reduction in quality of life for those suffering from such a disorder [12]. Indeed, these patients can no longer enjoy the simple pleasure of eating, as sometimes they need to have immediate access to a toilet facility. However, therapeutic treatment of such pathologies is rarely successful. Multiple factors can be considered to explain the relative lack of research into the causes and treatment of disturbed gastrointestinal functions, such as failure to identify disorders from patient-reported outcomes, and difficulties in pathophysiology, diagnosis, and drug development [13]. Therefore, researchers are struggling to find multiple ways to explore alternative therapy targeting the underlying mechanisms of gastrointestinal diseases. Remarkably, plant extracts constitute a diversified source of phytochemicals that can lead to the identification of novel compounds as alternative therapeutic agents to treat patients with intestinal disorders [14,15,16]. Traditionally, sweet potato is consumed to treat several illnesses, including diarrhea and stomach disorders. In addition, several studies have identified various phytochemicals from sweet potatoes. Within this line of research, our study on the activity of SPEs in the intestinal barrier led to the identification of a potential active compound, trifostigmanoside I, using a bioactivity-guided isolation technique.

In the epithelium, goblet cells release mucin, a polypeptide that protects the epithelial cells from invading pathogens and toxins [17]. The epithelial layer is considered the most important barrier against the external environment and selectively permits the absorption of electrolytes, water, and nutrients while defending the body against enteric flora, antigens, and toxins [17,18]. Our study revealed that trifostigmanoside I promoted mucin production, which ultimately led to enhanced protection of the epithelial layer to maintain intestinal barrier function. Several factors and various signaling pathways are involved in promoting mucin production, among which the activation of PKCα/β and its downstream molecule, ERK1/2 [7,8]. Our results revealed that trifostigmanoside I activated the phosphorylation of PKCα/β and ERK1/2 to promote mucin production and ultimately contributed in maintaining intestinal barrier function through active PKCα/β-ERK1/2 signaling. However, to explore other possible mediators of this effect, the inflammatory mechanism was also studied [19]. Tumor necrosis factor (TNF)-α is a key regulator of intestinal inflammation and is responsible for increasing the permeability of intestinal TJs. Activation of TNF-α leads to rapid activation of the NF-𝜅B/p65 signaling pathway [20,21]. Therefore, p65 is considered a biomarker of the activation of inflammation-related pathways affecting intestinal barrier function. For this reason, the effect of SPEs on LPS-induced phosphorylation of p65 was assessed. However, the results revealed that SPEs did not prevent LPS-mediated induction of p65 phosphorylation. This indicates that water SPE may not have the capability to inhibit or reduce the inflammatory pathway targeting p65 to maintain intestinal barrier function, or future study needs to be conducted to explore the anti-inflammatory property of water SPE.

TJs are formed by adjacent intestinal epithelial cells, and they are important for maintaining intestinal barrier function and for regulating the movements of various substances, including water, solutes, and ions, across the intestinal epithelium [22]. Several peripheral membranes and integral transmembrane proteins are involved in the regulation of TJ integrity. Indeed, the phosphorylation status, distribution, and expression levels of these proteins play a significant role in maintaining TJ barrier function. Moreover, these peripheral membrane proteins interact with other signaling proteins to regulate TJ integrity through different signal transduction pathways such as MAPK/ERK, myosin light chain protein kinase (MLCK), and protein kinase C (PKC) signaling [10,23]. In this study, since TS contributed to activation of ERK signaling, as shown in Figure 3A, TJ integrity was analyzed by measuring the expression of TJ proteins, Claudin-1, occluding, and ZO-1. However, there was no effect of water SPE on the expression of TJ proteins. Interestingly, water SPE partially rescued TJ damage, revealing that water SPE played a partially protective role against TJ damage from DSS-mediated damage, but further investigation is necessary to expose its mechanism. By exploring the active compounds from water SPE, we revealed that trifostigmanoside I was involved in maintaining the intestinal barrier function. Taken together, these results revealed that trifostigmanoside I, an active compound from water SPE, restored the function of MUC2 and partially protected the TJs through PKCα/β-ERK1/2 signaling to maintain the intestinal barrier function. However, more phytochemicals need to be explored to determine whether the effect of sweet potato is due to a single active compound or if more compounds are involved in the regulation of multiple signaling pathways implied in gastrointestinal disorders.

## Figures and Tables

**Table 1:** List of primers for RT- and semi-quantitative real-time PCR.

Primers	Sequences
Mucin 2 (MUC2)-F	5′-GTC TGC AAG TGC AAC ACC AG-3′
Mucin 2 (MUC2)-R	5′-GAG ACG GAC GAG ATG AGC TG-3′
ZO-1-F	5′-AAC GCT ATG AAC CCA TCC AG-3′
ZO-1-R	5′-CGG TTT GGT GGT CTG AAA GT-3′
Occludin-F	5′-GAA GCC AAA CCT CTG TGA GC-3′
Occludin-R	5′-GAA GAC ATC GTC AGG GGT GT-3′
Claudin 1-F	5′-CCG TTG GCA TGA AGT GTA TG-3′
Claudin 1-R	5′-CCA GTG AAG AGA GCC TGA CC-3′
GAPDH-F	5′-GGTGAAGGTCGGTGTGAACGGATTT-3′
GAPDH-R	5′-AATGCCAAAGTTGTCATGGATGACC-3′

## Data Availability

The data that support the findings of this study are available from the corresponding author upon reasonable request.

## Supplementary Materials

The following are available online at https://www.mdpi.com/1422-0067/22/1/291/s1.
