# Supplementary material for: Trifostigmanoside I, an Active Compound from Sweet Potato, Restores the Activity of MUC2 and Protects the Tight Junctions through PKCα/β to Maintain Intestinal Barrier Function
Source: Int J Mol Sci. 2020 Dec 30;22(1):291. doi: 10.3390/ijms22010291 (PMC7794767; doi:10.3390/ijms22010291)
Supplement: Supplementary file 1 [file ijms-22-00291-s001.pdf]

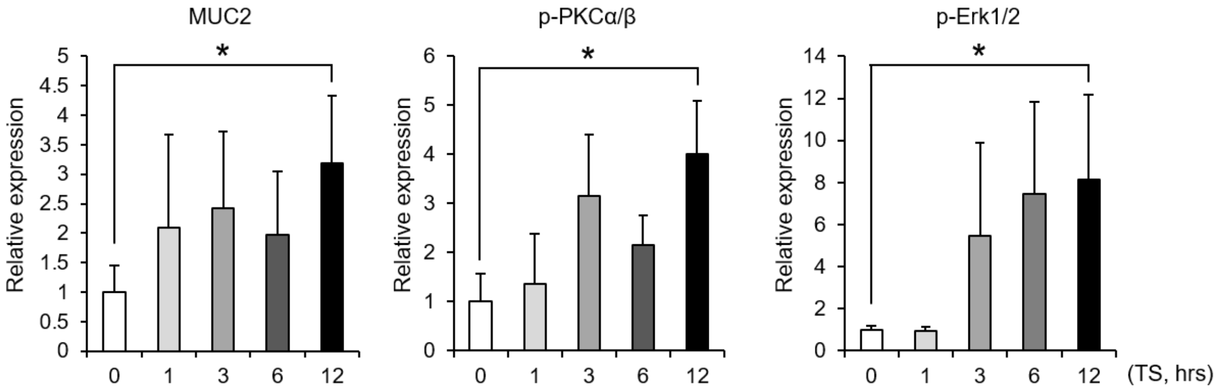

Supplementary Figure 1. Quantification of western blot results (Fig. 3A). Western blot results of MUC2, p-PKCα/β and p-Erk1/2 in TS-treated SL174T cells were quantified by using Image J program and statistical analysis was performed by using GraphPad Prism 7.04. *p<0.05.

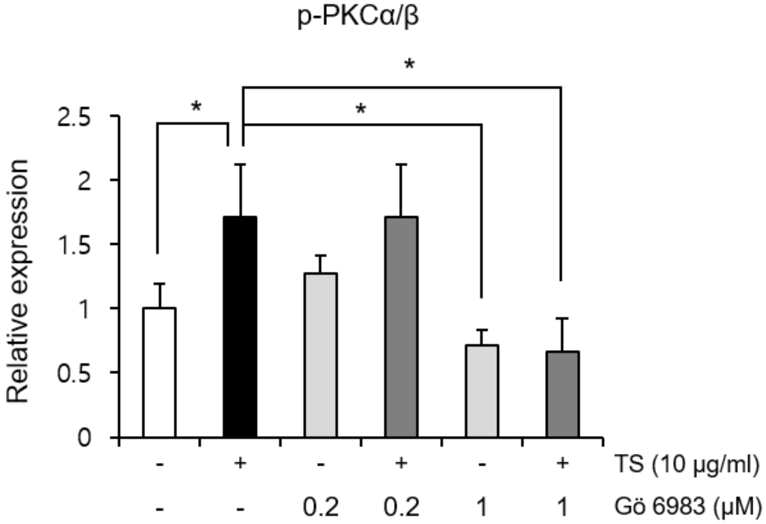

Supplementary Figure 2. Quantification of western blot results (Fig. 4A). Western blot results of p-PKCα/β in TS±Gö6983-treated LS174T cells were quantified by using Image J program and statistical analysis was performed by using GraphPad Prism 7.04. *p<0.05

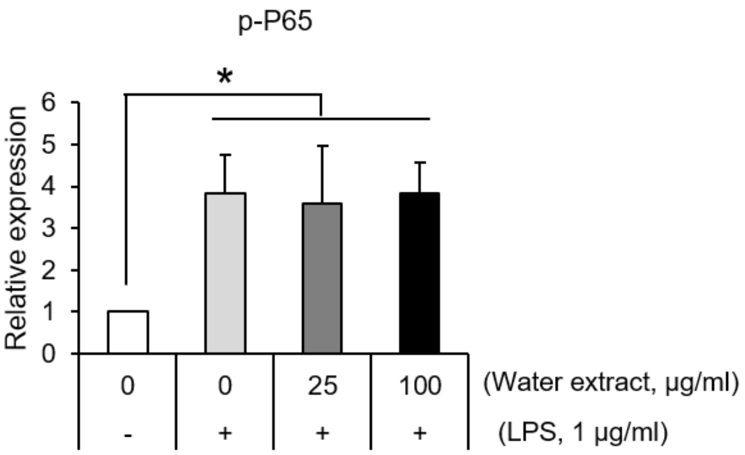

Supplementary Figure 3. Quantification of western blot results (Fig. 5). Western blot results of p-P65 in water extract-treated Raw264.7 cells were quantified by using Image J program and statistical analysis was performed by using GraphPad Prism 7.04. *p<0.05

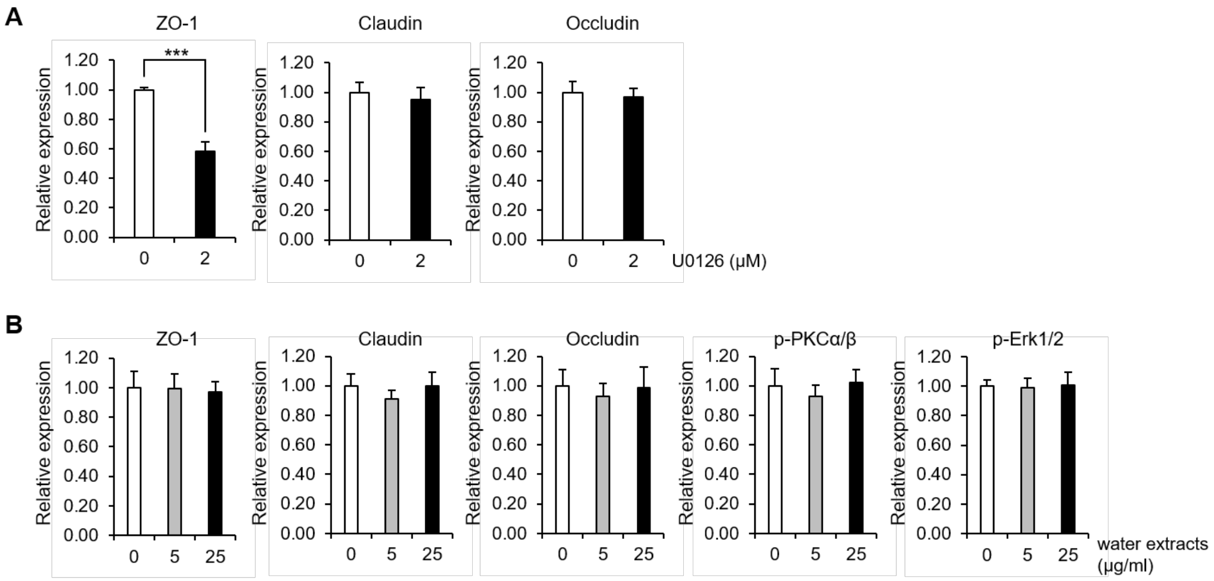

Supplementary Figure 4. Quantification of western blot results (Fig. 6A, B). Western blot results of tight junction proteins, p-PKCα/β and p-Erk1/2 in water extract-treated Raw264.7 cells were quantified by using Image J program and statistical analysis was performed by using GraphPad Prism 7.04.
